# BMI trajectory in childhood is associated with asthma incidence at young adulthood mediated by DNA methylation

**DOI:** 10.1186/s13223-021-00575-w

**Published:** 2021-07-23

**Authors:** Rutu Rathod, Hongmei Zhang, Wilfried Karmaus, Susan Ewart, Latha Kadalayil, Caroline Relton, Susan Ring, S. Hasan Arshad, John W. Holloway

**Affiliations:** 1 Division of Epidemiology, Biostatistics and Environmental Health, School of Public Health, University of Memphis, Memphis, TN USA; 2 College of Veterinary Medicine, Michigan State University, East Lansing, MI USA; 3 Human Development and Health, Faculty of Medicine, University of Southampton, Southampton, UK; 4 MRC Integrative Epidemiology Unit, University of Bristol, Bristol, UK; 5 Population Health Sciences, Bristol Medical School, University of Bristol, Bristol, UK; 6 National Institute for Health Research Bristol Biomedical Research Centre, University of Bristol and University Hospitals Bristol NHS Foundation Trust, Bristol, UK; 7 Clinical and Experimental Sciences, Faculty of Medicine, University of Southampton, Southampton, UK; 8 David Hide Asthma and Allergy Research Centre, Isle of Wight, UK; 9 NIHR Southampton Biomedical Research Centre, University Hospital Southampton, Southampton, UK

**Keywords:** Asthma acquisition, ALSPAC, BMI trajectory, DNA methylation, IOWBC

## Abstract

**Purpose:**

Body mass index (BMI) is associated with asthma but associations of BMI temporal patterns with asthma incidence are unclear. Previous studies suggest that DNA methylation (DNAm) is associated with asthma status and variation in DNAm is a consequence of BMI changes. This study assessed the direct and indirect (via DNAm) effects of BMI trajectories in childhood on asthma incidence at young adulthood.

**Methods:**

Data from the Isle of Wight (IoW) birth cohort were included in the analyses. Group-based trajectory modelling was applied to infer latent BMI trajectories from ages 1 to 10 years. An R package, *ttscreening*, was applied to identify differentially methylated CpGs at age 10 years associated with BMI trajectories, stratified for sex. Logistic regressions were used to further exclude CpGs with DNAm at age 10 years not associated with asthma incidence at 18 years. CpGs discovered via path analyses that mediated the association of BMI trajectories with asthma incidence in the IoW cohort were further tested in an independent cohort, the Avon Longitudinal Study of Children and Parents (ALSPAC).

**Results:**

Two BMI trajectories (high vs. normal) were identified. Of the 442,474 CpG sites, DNAm at 159 CpGs in males and 212 in females were potentially associated with BMI trajectories. Assessment of their association with asthma incidence identified 9 CpGs in males and 6 CpGs in females. DNAm at 4 of these 15 CpGs showed statistically significant mediation effects (p-value < 0.05). At two of the 4 CpGs (cg23632109 and cg10817500), DNAm completely mediated the association (i.e., only statistically significant indirect effects were identified). In the ALSPAC cohort, at all four CpGs, the same direction of mediating effects were observed as those found in the IoW cohort, although statistically insignificant.

**Conclusion:**

The association of BMI trajectory in childhood with asthma incidence at young adulthood is possibly mediated by DNAm.

**Supplementary Information:**

The online version contains supplementary material available at 10.1186/s13223-021-00575-w.

## Introduction

The epidemic of obesity continues to increase globally despite its increasing awareness [1]. Obesity increases the risk of many chronic diseases among children and adults [1]. Asthma is a common chronic respiratory condition that predominantly originates in early childhood [2]. Several longitudinal epidemiological studies have identified obesity as a major risk factor for asthma [3]. A dose–response relationship of elevated body mass index (BMI) on asthma incidence has been demonstrated in a meta-analysis of prospective epidemiologic studies [4]. In the course of puberty, a gender reversal in asthma prevalence has been observed with a higher prevalence among boys before puberty and a higher prevalence among girls after puberty [5, 6].

Many recent studies have suggested a role of epigenetic programming in relation to both obesity [7] and asthma [8]. One of the most widely studied epigenetic mechanisms is DNA methylation (DNAm), which is known to respond to environmental exposures. DNAm is a potentially reversible process where a methyl group is attached to a nucleotide and can result from both genetic and environmental factors. DNAm at specific cytosine-phosphate-guanines (CpG) sites has been found to be associated with BMI [9, 10] and asthma [11, 12]. Recent investigations have suggested that changes in DNAm in blood and in adipose tissue are primarily the consequence of BMI [13, 14] rather than the other way around.

**Fig. 1:**
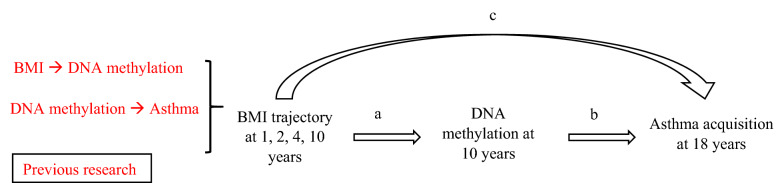
Path analyses assessing DNAm mediation effect on the association of BMI trajectory and asthma incidence. **a** Effects of BMI-trajectories on methylation of CpGs, controlled for secondhand smoking status at 1, 2 and 4 years. **b** Effects of CpGs on the incidence of asthma, controlled for BMI trajectories, socio-economic status (SES), active smoking status at 18 years, pubertal events (age at onset of voice deepening in males and age at onset of menarche in females). **c** Direct effects of BMI trajectories on asthma acquisition

We have previously demonstrated that subjects with high BMI over time have a higher risk of asthma [15]. Given that increased BMI is associated with asthma, and both these chronic conditions are associated with DNAm, we hypothesized that DNAm mediates the association of BMI trajectories before adolescence with asthma incidence in young adulthood (Fig. 1). Path analyses were utilized to examine the mediation effects of DNAm. There is heterogeneity regarding the role of sex in the obesity-asthma relationship among studies reporting asthma incidence by sex; some studies showed significant associations between obesity and asthma regardless of sex [16, 17], while others demonstrated associations only in males [18, 19] or only in females [20–22]. Thus, in this study, we stratify the analyses by sex in order to focus on the assessment of epigenetic mediation effects.

## Methods

### Study population

A population birth cohort was established on the Isle of Wight (IoW), UK, to prospectively study the natural history of allergic diseases among children. Of the 1536 pregnancies between January 1, 1989, and February 28, 1990, parents of all infants born over this period were contacted at birth, and subsequently, 1456 infants were enrolled following informed consent and exclusion. Follow‐ups for survey and clinical data were conducted at ages 1, 2, 4, 10, and 18 years. The IoW birth cohort (IOWBC) has been described in detail elsewhere [23]. Detailed interviews and examinations were completed for each child at each follow-up.

### Data collection—outcome, exposures, covariates

The International Study of Asthma and Allergy in Childhood (ISAAC) questionnaire was used to obtain information regarding asthma at 18 years [24]. The questions used to assess asthma were: ‘History of physician diagnosed asthma?’, ‘Wheezing or whistling in the chest in the last 12 months?’ and ‘Asthma treatment in the last 12 months?’ Based on responses to these questions, a participant was determined to have asthma if she/he had experienced recurrent wheezing in the last 12 months and been given a clinical diagnosis of asthma by the physician with or without being treated with asthma medications.

Asthma incidence at 18 years was the outcome of interest for this study and was defined as not having asthma by age 10 years but developed asthma by age 18 years (no → yes). Subjects with asthma at ages 1, 2, 4 or 10 years were excluded to focus on the association of persistent BMI patterns in childhood with young adult asthma incidence. Height and weight of each participant was assessed at ages 1, 2, 4, and 10 years. Body mass index (BMI) was calculated using weight in kilograms divided by height in meters-squared at each age. Information regarding sex, maternal smoking during pregnancy, duration of breastfeeding, maternal and paternal disease status of asthma and age at specific pubertal events, i.e., age at onset of voice deepening in males and age at onset of menarche in females, was extracted from questionnaire data. Socio-economic status (SES) was defined based on household income, number of rooms and maternal education. Active smoking status at 18 years was recorded as either never smoker, current smoker or past smoker. Second-hand smoking exposure was determined using information obtained for tobacco smoke exposure from mother, father, or others at ages 1, 2, and 4 years.

### DNA methylation

DNA was extracted from whole blood samples collected at 10 years of age using a standard salting out procedure [25]. One microgram of DNA was bisulfite-treated for cytosine to thymine conversion using the EZ 96-DNA methylation kit (Zymo Research, Irvine, CA, USA) for each sample, following the manufacturer’s standard protocol. Genome-wide DNAm for each CpG was assessed using either Illumina Infinium HumanMethylation450 BeadChips or the Methylation EPIC BeadChip (Illumina, Inc, San Diego, CA, USA), which interrogate > 484,000 and > 850,000 CpG sites, respectively. Arrays were processed using a standard protocol as described elsewhere [26], with multiple identical control samples assigned to each bisulfite conversion batch to assess assay variability. The BeadChips were scanned using a BeadStation, and the methylation level (beta (β) value) was calculated for each queried CpG locus using Methylation module of BeadStudio software.

DNAm data were preprocessed using the CPACOR pipeline for data from both platforms (HumanMethylation450 and MethylationEPIC) [27]. Specifically, the DNAm intensity data were quantile‐normalized using the R package, *minfi* [28]. Beta values were calculated representing proportions of intensity of methylated (*M*) over the sum of methylated and unmethylated (*U*) sites/probes (*β* = *M*/ [c + *M* + *U*], where c is a constant to prevent zero in the denominator if *M* + *U* is too small). Beta values close to 0 or 1 tend to suffer from severe heteroscedasticity, and it has been demonstrated that base-2 logit transformed beta values (denoted as M-values) perform better in differential analysis of methylation levels [29]. Therefore, M-values were used to represent methylation levels in the analysis.

Principal components (PCs) inferred based on control probes to represent latent chip‐to‐chip and technical variations were generated. Since DNAm data were from two different platforms, PCs were determined based on DNAm at shared control probes. In total, 195 control probes were shared between the two platforms (450 K and EPIC) and used to calculate the control probe PCs and the top 15 were used to represent latent batch factors [27]. These 15 PCs were included in subsequent analyses. Probes not reaching a detection p-value of 10^–16^ in at least 95% of samples were excluded. A comparable criterion was applied to exclude samples with a low quality of DNAm measurement. CpGs on the sex chromosomes were excluded to avoid bias in our analyses. Probes that contained single nucleotide polymorphisms (SNPs) within 10 base pairs of a targeted CpG site with a minor allele in at least 0.7% subjects (corresponding to at least 10 subjects in IoW with n = 1456) were excluded due to their influence on DNAm. After preprocessing, a total of 442,475 CpGs in common between the two platforms (450 K and EPIC) were included in the analyses.

Since blood is a mixture of functionally and developmentally distinct cell populations [30], adjusting for cell type compositions potentially mitigates the possibility of confounding cell heterogeneities in DNAm measured from blood samples [31]. To this end, we estimated cell type proportions using the method proposed by Jaffe and Irizarry [32], adapted from Houseman et al. [33], using the Bioconductor *minfi* package [28]. The estimated cell type proportions of CD4 + T cells, natural killer cells, neutrophil, B cells, monocytes, and eosinophil cells were included in the analyses as confounding factors.

### Genome-wide RNA-seq gene expression data generation

Gene expression levels from peripheral blood samples collected at 26 years from the IOWBC was determined using paired-end (2 × 75 bp) RNA sequencing with the Illumina Tru-Seq Stranded mRNA Library Preparation Kit with IDT for Illumina Unique Dual Index (UDI) barcode primers following the manufacturer’s recommendations. All samples were sequenced twice using the identical protocol and for each sample the output from both runs were combined. FASTQC was run to assess the quality of the FASTQ files (https://www.bioinformatics.babraham.ac.uk/projects/fastqc/). Reads were mapped against Human Genome (GRch37 version 75) using HISAT2 (v2.1.0) aligner [34]. The alignment files, produced in the Sequence Alignment Map (SAM) format, were converted into the Binary Alignment Map (BAM) format using SAMtools (v1.3.1) [35]. HTseq (v0.11.1) was used to count the number of reads mapped to each gene in the same reference genome used for alignment [36]. Normalized read count FPKM (Fragments Per Kilobase of transcript per Million mapped reads) were calculated using the countToFPKM package (https://github.com/AAlhendi1707/countToFPKM) and the log-transformed values were used for data analysis.

### Statistical analysis

To examine whether the analytic sample (n = 224) reasonably represents the complete cohort (n = 1456), chi-square tests for categorical variables and one-sample t-tests for continuous variables were applied, stratified by sex. In addition, all the subsequent analyses were stratified by sex, considering gender reversal in asthma prevalence.

### BMI trajectories

BMI trajectories were determined separately for both the sexes using their BMI values at ages 1, 2, 4 and 10 years. Our study focused on temporal patterns of BMI. Thus, the less informative standardized BMI (or Z-BMI) was not needed [37]. A group-based trajectory modelling, also referred to as a semiparametric mixture model [38, 39], was applied using PROC TRAJ in SAS [40] to identify BMI developmental paths in the form of trajectories across ages 1, 2, 4 and 10 years. The group-based trajectory method presumes that the data comprises of latent distinct groups (trajectories) that best summarize the distinct features as parsimonious as possible [38, 39]. Models with one to three groups were estimated for linear and quadratic terms. The selection of a best fit model was based on the smallest Bayesian Information Criterion value. Individuals were assigned to one of the trajectories/groups based on their highest estimated group-membership probabilities.

### Association of BMI trajectories with asthma incidence

Subjects with asthma at ages 1, 2, 4 and 10 years were excluded from the analysis. We used multivariable logistic regression to evaluate the association of BMI-trajectory (independent variable) with asthma incidence at 18 years (dependent variable) along with covariates and confounders potentially associated with asthma incidence in the model: SES, active smoking status at 18 years, height at 10 years, maternal smoking during pregnancy, duration of breastfeeding (in weeks), parental history of asthma and age at pubertal events. For males, age at voice deepening, and for females, age at menarche were included in the model.

### Screening for CpGs related to BMI trajectories

We regressed the M-values of DNAm at each CpG site on the aforementioned 15 PCs obtained from control probes and the 6 cell type proportions [33] to obtain batch- and cell-type-adjusted DNAm (residuals) for each sex. These residuals were batch- and cell types-adjusted DNAm and used in subsequent analyses. We applied an R package, *ttScreening*, to screen CpGs at 10 years with DNAm potentially associated with BMI trajectory groups [41]. In the selection process, the method implemented in the package utilizes training and testing data in robust linear regressions. The screening was performed separately for each sex. Following the guideline [41], the minimum frequency of selecting an informative CpG sites was set at 50%, i.e., a CpG site gained statistical significance in at least 50% of the randomly selected training and testing data set pairs. For CpGs that passed the screening, they were treated as potential BMI-trajectory-associated-CpGs.

### Screening for BMI-trajectory-related CpGs associated with asthma incidence

Subjects with asthma at ages 1, 2, 4 and 10 years were excluded from the analysis. We used multivariable logistic regression to evaluate the association of potential BMI-trajectory-associated-CpGs (independent variable) with asthma incidence at 18 years (dependent variable) along with covariates and confounders potentially associated with asthma incidence in the model: BMI trajectory groups [42, 43], SES, active smoking status at 18 years, and age at pubertal events. For males, age at voice deepening, and for females, age at menarche were included in the model. Statistical significance for this in-depth screening process was set at 0.05.

### Path analyses

Using path analyses, we explored the association between BMI trajectories at 1, 2, 4, 10 years and asthma incidence at 18 years, and whether the relationship between these variables was mediated by DNAm at 10 years (Fig. 1), with potential confounders included in each path. Goodness of fit criteria using chi-square test p-value > 0.05, RMSEA < 0.05, CFI > 0.95 was used. The path coefficients (direct and indirect estimates) represent the partial correlation between the independent and dependent variables after adjusting for confounders and covariates used in the model above. An R package, *MplusAutomation*, was utilized to iteratively call *MPlus* from R to perform path analyses with each of the CpGs as a mediator (Fig. 1) [44].

### Association of DNAm with gene expression

To evaluate the biological relevance of the identified mediating CpGs, association between DNAm (in M-values) and expression of genes within a 500 kilo base pairs (kbps) window (250kbps upstream and 250kbps downstream of the CpG site) was evaluated at 26 years using linear regressions. Gene expression (n = 140) was the dependent variable, and DNAm was the independent variables.

### Replication cohort—the Avon Longitudinal Study of Children and Parents (ALSPAC) cohort

CpGs shown to mediate the association of BMI trajectory groups with asthma incidence in the IOWBC were further assessed in an independent cohort, the Avon Longitudinal Study of Children and Parents (ALSPAC) [45, 46]. Women residing in the South West of England who were pregnant and expecting to deliver between April 1, 1991 and December 31, 1992 were eligible to be recruited. Of the 14,541 pregnant women eligible for recruitment, 13,761 were included in the study with 10,321 participants having their DNA sampled. DNAm in the ALSPAC cohort was assessed using the Infinium HumanMethylation450 BeadChip. The pre-processing of DNAm was performed by correcting for batch effects using the *minfi* package [28] and removing CpGs with detection p-value ≥ 0.01. Samples were flagged that contained sex-mismatch based on X-chromosome methylation. Estimated cell type proportions of CD4 + T cells, natural killer cells, CD8 + T cells, B cells, monocytes, and granulocytes cells were used in the analyses to adjust for cell heterogeneity. DNAm at 7 years was included in our study and its residuals were calculated by regressing M-values on cell type proportions. Please note that the study website contains details of all the data that is available through a fully searchable data dictionary and variable search tool (http://www.bristol.ac.uk/alspac/researchers/our-data/).

BMI trajectories were modeled at ages 1, 2, 4 and 7 years separately in both sexes. Subjects with asthma at 7 and 10 years were excluded, and asthma incidence was assessed at 17 years. Identical path analysis models as those applied in the IoW cohort were used with comparable covariates available in ALSPAC, including SES, active smoking status at 17 years and pubertal events. Secondhand smoking at 7 years was not considered due to low counts in asthma incidence in one of the secondhand smoking categories.

For the CpGs showing mediation effects, the genes annotated to the CpGs were summarized along with information such as gene location, chromosome number based on Illumina's manifest file and SNIPPER (https://csg.sph.umich.edu/boehnke/snipper/) version 1.2.

## Results

**Fig. 2:**
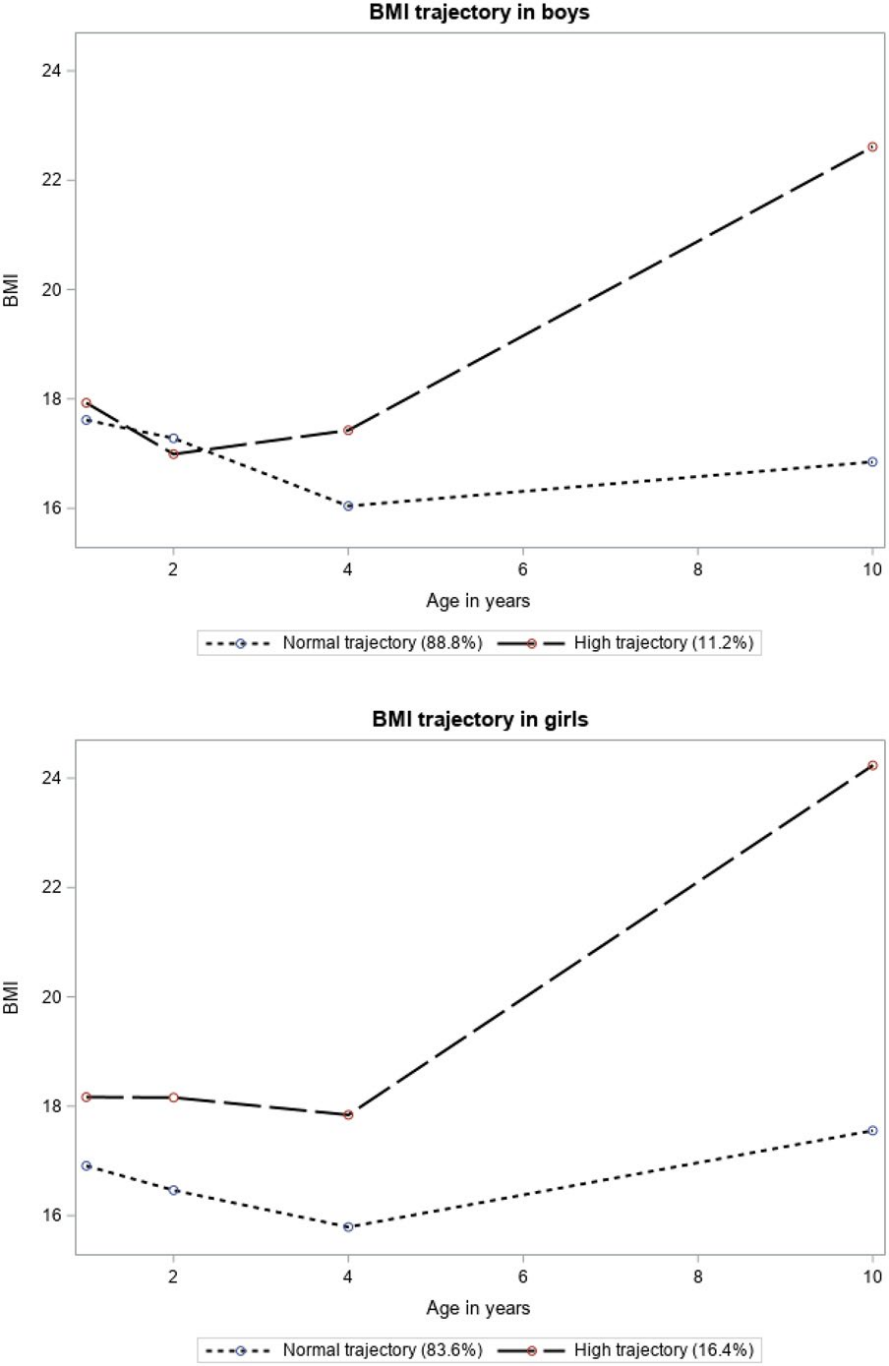
BMI trajectories across first 10 years of life in boys and girls respectively in IoW

We estimated BMI trajectory based on data from subjects with BMI available for at least two time points. In total, BMI of 602 boys and of 577 girls were included in the trajectory analyses. Two BMI trajectories were identified for both sexes that best summarized the complex developmental course of BMI across the first 10 years of life (Fig. 2) by optimizing the Bayesian Information Criterion. Since one trajectory included subjects potentially overweight or obese [47], we labelled that trajectory as a ‘high’ BMI trajectory, and the other as a ‘normal’ BMI trajectory. The distribution of variables used in the analysis is shown by the BMI trajectory groups in Additional file 1: Table S1. Results from logistic regressions indicated that subjects with high BMI trajectory had significantly increased odds of asthma incidence at 18 years in males (OR = 8.27, p-value = 0.004) and females (OR = 4.89, p-value = 0.001) after adjusting for confounders and covariates.

The end point that we focused on in this study was asthma acquisition at, or close to, post-adolescence. Thus, in IOWBC, of the 1456 subjects in the cohort, 320 subjects had asthma at or before age 10 years and were excluded from the study. Of the remaining 1136 subjects, 122 male and 102 female participants also had DNAm data at 10 years and were included in the path analyses. Descriptive statistics indicated that the analytical subsamples represented the complete IoW birth cohort for all variables except for second-hand smoking in males (Table 1).

**Table 1:** Comparison of analytical subsample with complete cohort

Variables		Males			Females		
Categorical variables		Subsample (n = 122); n (%)	Complete cohort (n = 552); n (%)	P-value	Subsample (n = 102); n (%)	Complete cohort (n = 584); n (%)	P-value
Asthma incidence	Yes	12 (9.84%)	31 (7.43%)	0.39	8 (7.84%)	45 (9.59%)	0.58
	No	110 (90.16%)	386 (92.57%)		94 (92.16%)	424 (90.41%)	
Socio-economic status	Low	15 (12.40%)	75 (16.09%)	0.38	18 (17.82%)	78 (15.06%)	0.77
	Mid	97 (80.17%)	345 (74.03%)		75 (74.26%)	400 (77.22%)	
	High	9 (7.44%)	46 (9.87%)		8 (7.92%)	40 (7.72%)	
Active smoking status (18 years)	Past	28 (23.14%)	76 (17.55%)	0.37	21 (20.79%)	100 (20.37%)	0.55
	Current	28 (23.14%)	112 (25.87%)		22 (21.78%)	132 (26.88%)	
	Never	65 (53.72%)	245 (56.58%)		58 (57.43%)	259 (52.75%)	
Second-hand smoking (1, 2, 4 years)	Yes	62 (52.10%)	333 (62.95%)	0.03	58 (56.86%)	337 (59.86%)	0.57
	No	57 (47.90%)	196 (37.05%)		44 (43.14%)	226 (40.14%)	

Continuous variables		Mean ± SD	Mean ± SD		Mean ± SD	Mean ± SD	
Age of puberty		14.14 ± 1.10	14.26 ± 1.17	0.25	12.6 ± 1.35	12.7 ± 1.41	0.54

To identify candidate CpGs potentially associated with BMI trajectory groups, *ttScreening* was applied to the 442,475 CpGs stratified for sex using residuals (batch- and cell-type-adjusted DNAm) at 10 years of age. In total, 159 CpGs in males and 212 CpGs in females passed screening. These CpGs were treated as potentially BMI trajectory associated CpGs and were included in the subsequent analyses.

**Fig. 3:**
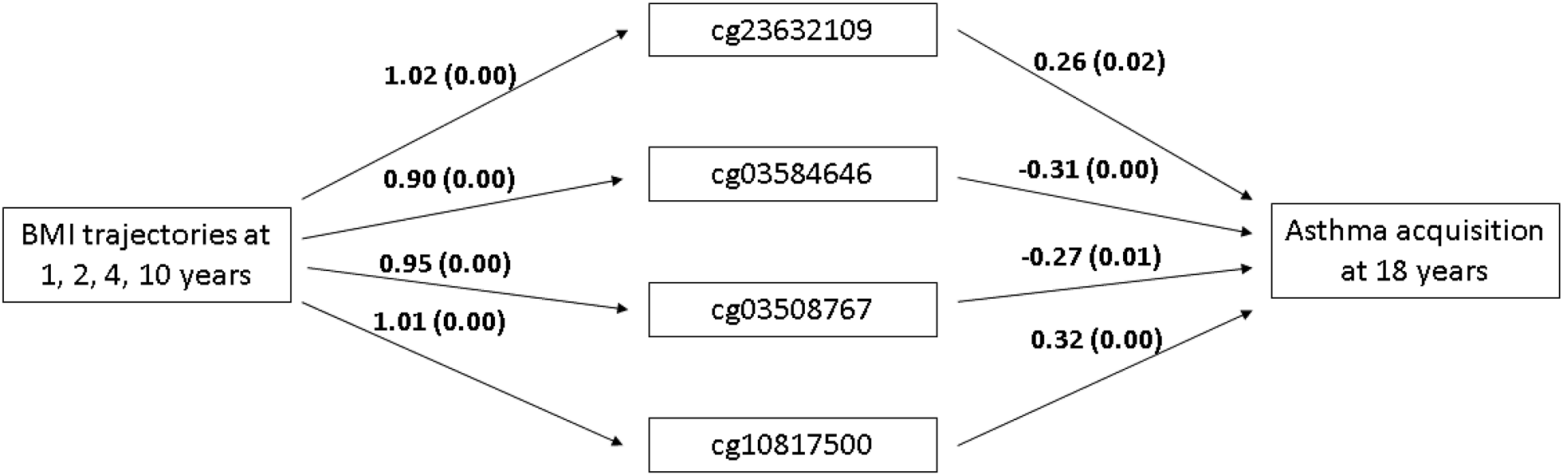
Indirect effects of childhood BMI trajectory on adulthood asthma incidence via DNAm at four CpGs. The figure shows the estimates (and p-values) of direct effects at each path, based on which indirect effects of BMI trajectory were inferred. For instance, the coefficient of 1.02 indicates that the methylation at cg23632109 in the high BMI trajectory group is 1.02 higher on average compared to the DNAm in the normal BMI trajectory group. The indirect effect of BMI trajectory via cg23632109 is obtained by 1.02*0.26 = 0.27 (Table 2). Goodness of fit criteria: Chi-square test p-value > 0.05, RMSEA < 0.05, CFI > 0.95 (except cg03584646: chi-sq. < 0.05, RMSEA = 0.15, CFI = 0.78). The first CpG is for males and the remaining three CpGs are for females

For each CpG that passed the screening, its association with asthma incidence at age 18 years was further evaluated. After controlling for potential confounders, at significance level of 0.05, nine CpGs in males and six CpGs in females were found to be associated with asthma incidence at 18 years. These CpGs were tested for their mediation effects of BMI trajectories at ages 1, 2, 4, 10 years on asthma incidence at 18 years using path analysis. One CpG in males and three CpGs in females showing such mediation effects were identified (Table 2). At two of the four CpGs (cg23632109 in males and cg10817500 in females), BMI trajectory only showed indirect effects on the risk of asthma incidence via DNAm at these two CpG sites, and no statistically significant direct effects were observed. To help understand the mediating effects of DNAm, Fig. 3 included direct effects for each path at each CpG site. At cg23632109 in males and at cg10817500 in females, the coefficients suggest that high BMI trajectory is associated with high DNAm, which was further linked to an increased risk of asthma incidence at 18 years (Fig. 3). For the other two CpGs, cg03584646 and cg03508767 in females, both direct and indirect effects are statistically significant (Table 2). In particular, with direct effects of BMI trajectory on asthma incidence, high BMI trajectory was associated with increased risk of asthma incidence at 18 years, but such an association was attenuated by DNAm at cg03584646 and cg03508767 in that subjects with high BMI trajectory tended to have higher DNAm at these two loci, which was further associated with lower risk of asthma.

**Table 2:** Effects of childhood BMI trajectories on asthma incidence in adulthood via pre-adolescence DNAm

CpG sites	IoW^a^				ALSPAC^b^				Genes	Gene location	Chr.^e^
	Indirect eff.^c^	P-value	Direct eff.^d^	P-value	Indirect eff.^c^	P-value	Direct eff.^d^	P-value			
cg23632109	0.27	0.03	0.06	0.89	**0.02**	0.92	0.53	0.37	*TBC1D16*	5′UTR, 3′UTR	17
cg03584646	− 0.28	0.04	1.01	0.01	− **0.09**	0.60	0.64	0.48	*TBC1D8*	Body, 3′UTR	2
cg03508767	− 0.25	0.04	0.99	0.01	− **0.36**	0.23	0.83	0.31	*RASA2*	3′UTR	3
cg10817500	0.32	0.02	0.39	0.34	**0.09**	0.87	0.48	0.65	*SSH1*	5′UTR, 1st Exon,	12

The first CpG is for males and the remaining three CpGs are for females. The indirect effects of CpGs in IoW and ALSPAC cohort in the same direction are highlighted in bold font.

^a^For the analyses in IoW, the path analyses were adjusted for second-hand smoking status at 1, 2 and 4 years, socio-economic status (SES), active smoking status at 18 years, age of pubertal events (age at onset of voice deepening for males and age at onset of menarche for females)

^b^Analyses of ALSPAC used similar covariates: active smoking status at 17 years, socio-economic status (SES) and pubertal events

^c^Indirect eff.: estimates of indirect effects

^d^Direct eff.: estimates of direct effects

^e^Chr.: Chromosome

The association of the identified mediating CpGs and expression of the neighboring mapped genes was also evaluated. Of the 4 identified CpGs, significant associations were observed at 3 CpGs with 5 genes (Table 3). At 3 of the 5 genes, the relationship was negatively correlated, such that a higher DNAm was associated with lower expression levels for the respective genes. For instance, in females, a one unit increase in DNAm levels of cg03508767 was followed by a downregulation of 0.41 units in the *PCDH1* gene.

**Table 3:** Association of DNAm with expression of neighboring mapped genes

CpG site	Gene name	Estimate	P-value	Approximate distance between CpG site and gene (in k bps)	Sex
cg23632109	*HSBP1L1*	0.41	0.028	166–173	Males
cg03508767	*PCDH1*	**− **0.41	0.030	73–99	Females
	*TAS2R5*	**− **0.55	0.037	157–159	Females
cg10817500	*LINC0022*	**− **0.55	0.025	166–179	Females
	*SULT1C4*	0.45	0.032	248–249	Females

No neighboring genes of cg03584646 showed association with DNAm.

To assess reproducibility, these four CpGs were further tested in the ALSPAC cohort. Similar to IOWBC, BMI trajectories were formulated in ALSPAC cohort using BMI at 1, 2, 4 and 7 years (Additional file 1: Figure S1). At all the 4 CpG sites, direction of indirect and direct effects were consistent with those identified in the IoW cohort, although none of the indirect effects were statistically significant at the level of 0.05 (Table 2). These four CpGs were mapped to four nearest genes (Table 2).

## Discussion

We assessed the direct and indirect effects of childhood BMI trajectory on post-adolescence asthma incidence via DNAm. Two BMI trajectories, high BMI trajectory and normal BMI trajectory, were identified in both the discovery cohort (IOWBC) and the replication cohort (ALSPAC). The two trajectories were similar at early ages and as the children grew, the difference between trajectories increased, and some children were overweight or obese by 10 years and hence belonged in the high BMI trajectory. In the IOWBC, DNAm at four CpGs was shown to mediate the association of BMI trajectories with asthma incidence at age 18 years. At two of the four CpGs (i.e., cg23632109 in males and cg10817500 in females), only indirect effects of BMI trajectory on asthma incidence were observed and high BMI trajectory was positively associated with high risk of asthma incidence via high DNAm. It is also interesting to note that CpGs showing indirect effects were unique between males and females, which might be due to potentially different underlying mechanisms of asthma incidence between the two sexes. At the other two CpGs (cg03584646 and cg03508767), the total effects, encompassing of both direct and indirect effects, remain consistent with existing literature such that subjects with a high BMI trajectory had greater odds of asthma incidence [15]. Nevertheless, the strength of this association was attenuated at these two sites. Although the longitudinal study design could not prove causality, we postulate that these two CpGs may have a possibility of being protective and further investigations on such a postulation are needed. The same direction of mediation effects were observed at all the four CpGs in ALSPAC, although they were not statistically significant.

Assessment of biological relevance of the identified mediating CpGs indicated a potential epigenetic regulatory functionality of these CpGs on expression of their neighboring genes. Previous research has shown that common genetic variations in *PCDH1* gene increases the risk of developing asthma [48] and bronchial hyperresponsiveness [49]. It has also been shown that polymorphisms in TAS2Rs (*TAS2R5*) may predict outcomes and therapeutic responses in asthmatic individuals [50]. Given our findings, there is a possibility that correlation of the identified CpGs corresponding to these two genes with gene expression may have been due to genetic effects via methylation quantitative trait loci (methQTL) [51, 52] and further in-depth assessment is certainly warranted.

Three of the four identified genes (Table 2), *TBC1D16, TBC1D8, and RASA2,* have been previously implicated in relation to asthma and/or obesity/BMI, supporting the informativity of genes identified in this study. For example, the interleukin-6 receptor (*IL-6R*) is linked to increased risk of asthma [53] and obesity [54], and its expression is positively associated with expression of *TBC1D8* and negatively with expression of *TBC1D16* [55]*.* One study noted that DNAm of *TBC1D8* was associated with obesity [56], while others showed that expression of *TBC1D8* was positively associated with obesity in abdominal and gluteal subcutaneous adipose tissue [57]. Many studies have suggested a connection between *RASA2* and BMI [58, 59], as well as its association with atopy and asthma [60]. While these previous studies have focused on the association between gene expression and/or genetic variation in these genes with BMI and asthma, the results of this study, from the angel of epigenetics, suggest that these genes may act as a potential epigenetic mediator between BMI trajectories and asthma.

Adolescence is a period in which both males and females experience rapid growth and in which there are clear sex-specific changes in asthma incidence. The availability of asthma status at two key time points, pre- and post-adolescence, offered us the opportunity to examine asthma incidence during this critical period. In comparison to measuring BMI at discrete time points, BMI trajectories allow for the dynamic visualization of BMI changes over time for certain groups of participants and allow researchers to follow similar developmental patterns of BMI over age, thereby reflecting unique features of each group. The use of BMI trajectories allows for the simultaneous consideration of intensity, age of onset and duration of adiposity, which may improve the predictability of future BMI patterns. To our knowledge, this is the first study to examine the DNAm at pre-adolescence mediates the association of BMI trajectories in childhood with asthma incidence at young adulthood. This study design with a unique time order, encapsulating pre-adolescence to post-adolescence, allowed for the dissection of the total effects of BMI trajectory on the risk of asthma and whether and how DNAm affects this relationship.

The direction of direct and mediating effects was consistent between the two cohorts at all four CpG sites identified in the IoW cohort. Statistical significance, however, was not observed at those CpG sites in the ALSPAC cohort. One possible reason for the lack of statistical significance in the ALSPAC cohort might be due to the differences in the ages of assessment for both asthma and BMI between ALSPAC and the IoW cohort. In ALSPAC, BMI up to age 7 years was included in trajectory analyses and DNAm was assessed at age 7 years, while in IOWBC, it was at age 10 years (up to age 10 years BMI and age 10 years DNAm). It is possible that underlying epigenetic mechanisms at age 7 years were not strong enough to be detected. On the other hand, it has been argued whether statistical significance is more important than clinical significance in the situation that our data could not satisfy both [61]. In terms of marker detection, we feel agreement in clinical significance is more important, i.e., the agreement in the direction of associations, since the behavior of those markers is linked to the risk of asthma incidence. CpGs with opposite directions of associations between the discovery and replication cohorts will lose a potential to serve as markers. In our study, at all the discovered CpGs, consistent directions of associations (both direct and indirect effects) were found in the replication cohort, ALSPAC. If this had happened by chance, the probability of this strong coincidence is 0.0039 (calculated by multiplication of the p-values for direct and indirect effects in ALSPAC, i.e., 0.92*0.37*0.60*0.48*0.23*0.31*0.87*0.65 = 0.0039). Such a low probability indicates the occurrence of this result being solely due to chance is very unlikely. Nevertheless, the statistically insignificant findings in the ALSPAC cohort indicate that caution is needed when generalizing the findings. Further assessment of these CpGs in large scale studies and different populations will benefit the validity and generalizability of the identified markers.

Some limitations are present in our study. The two BMI trajectories inferred in the IOWBC and ALSPAC were based on their best fit. Since both birth cohorts are population based, we expect the trajectories will reflect populations with similar features. On the other hand, demographics and age range in this study may be limiting factors in the external validity of findings, and hence generalization of these trajectories and related findings to other populations should be implemented with caution. In one of our earlier studies [15], four trajectories were identified, “normal”, “early persistent obesity”, “delayed overweight”, and “early transient overweight”, which had more detailed features compared to the two trajectories inferred in the current study. In light of our genome-scale analyses, in addition to a carefully planned screening process, to avoid power loss, we focused on parsimonious trajectories to detect epigenetic factors potentially associated with overweight or obesity in general. The CpGs included in the path analysis model were pre-selected based on their association with BMI trajectory and asthma incidence during the screening process. Because of these detailed considerations, in the discovery phase, multiple testing was not adjusted. However, large scale studies are certainly warranted to scrutinize the connection between epigenetics and detailed BMI trajectories. In addition, we evaluated the contributions of each CpG site. These CpGs may be correlated and jointly impact asthma incidence, which could not be addressed by the present study. Although future studies are warranted to further examine the credibility of the identified CpGs, the consistency in the results between the two cohorts indicates a role for epigenetics in the association of obesity and asthma. The identified CpGs have the potential to improve our understanding of the underlying biological pathways in the connection between obesity and asthma incidence during adolescence.

## Supplementary Information

**Additional file 1: Table S1.** Distribution of variables in each of the BMI trajectory groups. **Figure S1.** BMI trajectories across first 7 years of life in boys and girls respectively in ALSPAC.

## Data Availability

The datasets analyzed during the current study are available from the corresponding author on reasonable request for the IOWBC. For the ALSPAC data, please contact the ALSPAC executive committee (alspac-exec@bristol.ac.uk).

## References

[1] Mitchell NS, Catenacci VA, Wyatt HR, Hill JO (2011). Obesity: overview of an epidemic. Psychiatr Clin North Am.

[2] Gern JE, Lemanske RF, Busse WW (1999). Early life origins of asthma. J Clin Investig.

[3] Peters U, Dixon AE, Forno E (2018). Obesity and asthma. J Allergy Clin Immunol.

[4] Beuther DA, Sutherland ER (2007). Overweight, obesity, and incident asthma: a meta-analysis of prospective epidemiologic studies. Am J Respir Crit Care Med.

[5] Han YY, Forno E, Celedón JC (2020). Sex steroid hormones and asthma in a nationwide study of U.S. adults. Am J Respir Crit Care Med.

[6] Hohmann C, Keller T, Gehring U (2019). Sex-specific incidence of asthma, rhinitis and respiratory multimorbidity before and after puberty onset: individual participant meta-analysis of five birth cohorts collaborating in MeDALL. BMJ Open Respir Res.

[7] Ling C, Ronn T (2019). Epigenetics in human obesity and type 2 diabetes. Cell Metab.

[8] Yang IV, Schwartz DA (2012). Epigenetic mechanisms and the development of asthma. J Allergy Clin Immunol.

[9] Mendelson MM, Marioni RE, Joehanes R (2017). Association of body mass index with DNA methylation and gene expression in blood cells and relations to cardiometabolic disease: a mendelian randomization approach. PLoS Med.

[10] Ding X, Zheng D, Fan C (2015). Genome-wide screen of DNA methylation identifies novel markers in childhood obesity. Gene.

[11] Arathimos R, Suderman M, Sharp GC (2017). Epigenome-wide association study of asthma and wheeze in childhood and adolescence. Clin Epigenet.

[12] Reese SE, Xu CJ, den Dekker HT, Lee MK, Sikdar S, Ruiz-Arenas C, Merid SK, Rezwan FI, Page CM, Ullemar V, Melton PE, Oh SS, Yang IV, Burrows K, Söderhäll C, Jima DD, Gao L, Arathimos R, Küpers LK, Wielscher M, Rzehak P, Lahti J, Laprise C, Madore AM, Ward J, Bennett BD, Wang T, Bell DA; BIOS consortium, Vonk JM, Håberg SE, Zhao S, Karlsson R, Hollams E, Hu D, Richards AJ, Bergström A, Sharp GC, Felix JF, Bustamante M, Gruzieva O, Maguire RL, Gilliland F, Baïz N, Nohr EA, Corpeleijn E, Sebert S, Karmaus W, Grote V, Kajantie E, Magnus MC, Örtqvist AK, Eng C, Liu AH, Kull I, Jaddoe VWV, Sunyer J, Kere J, Hoyo C, Annesi-Maesano I, Arshad SH, Koletzko B, Brunekreef B, Binder EB, Räikkönen K, Reischl E, Holloway JW, Jarvelin MR, Snieder H, Kazmi N, Breton CV, Murphy SK, Pershagen G, Anto JM, Relton CL, Schwartz DA, Burchard EG, Huang RC, Nystad W, Almqvist C, Henderson AJ, Melén E, Duijts L, Koppelman GH, London SJ. Epigenome-wide meta-analysis of DNA methylation and childhood asthma. J Allergy Clin Immunol. 2019;143(6):2062–74. 10.1016/j.jaci.2018.11.043. 10.1016/j.jaci.2018.11.043 PMC6556405 30579849

[13] Wahl S, Drong A, Lehne B (2017). Epigenome-wide association study of body mass index, and the adverse outcomes of adiposity. Nature.

[14] Reed ZE, Suderman MJ, Relton CL, Davis OSP, Hemani G (2020). The association of DNA methylation with body mass index: distinguishing between predictors and biomarkers. Clin Epigenet.

[15] Ziyab AH, Karmaus W, Kurukulaaratchy RJ, Zhang H, Arshad SH (2014). Developmental trajectories of body mass index from infancy to 18 years of age: prenatal determinants and health consequences. J Epidemiol Commun Health.

[16] Ford ES, Mannino DM, Redd SC, Mokdad AH, Mott JA (2004). Body mass index and asthma incidence among USA adults. Eur Respir J.

[17] Nystad W, Meyer HE, Nafstad P, Tverdal A, Engeland A (2004). Body mass index in relation to adult asthma among 135,000 Norwegian men and women. Am J Epidemiol.

[18] Huovinen E, Kaprio J, Koskenvuo M (2003). Factors associated to lifestyle and risk of adult onset asthma. Respir Med.

[19] Mannino DM, Mott J, Ferdinands JM (2005). Boys with high body masses have an increased risk of developing asthma: findings from the National Longitudinal Survey of Youth (NLSY). Int J Obes.

[20] Lampalo M, Majer M, Ferara N, Milošević M, Barišić Kutija M, Jukić I (2019). Gender differences in relationship between body mass index and asthma. Psychiatr Danub.

[21] Wang L, Wang K, Gao X, Paul TK, Cai J, Wang Y (2015). Sex difference in the association between obesity and asthma in US adults: findings from a national study. Respir Med.

[22] Willeboordse M, van den Bersselaar DL, van de Kant KD, Muris JW, van Schayck OC, Dompeling E (2013). Sex differences in the relationship between asthma and overweight in Dutch children: a survey study. PLoS ONE.

[23] Arshad SH, Holloway JW, Karmaus W (2018). Cohort profile: The Isle Of Wight whole population birth cohort (IOWBC). Int J Epidemiol.

[24] Asher MI, Keil U, Anderson HR (1995). International study of asthma and allergies in childhood (ISAAC): rationale and methods. Eur Respir J.

[25] Miller SA, Dykes DD, Polesky HF (1988). A simple salting out procedure for extracting DNA from human nucleated cells. Nucleic Acids Res.

[26] Bibikova M, Fan JB (2009). GoldenGate assay for DNA methylation profiling. Methods Mol Biol (Clifton, NJ).

[27] Lehne B, Drong AW, Loh M (2015). A coherent approach for analysis of the Illumina HumanMethylation450 BeadChip improves data quality and performance in epigenome-wide association studies. Genome Biol.

[28] Aryee MJ, Jaffe AE, Corrada-Bravo H (2014). Minfi: a flexible and comprehensive Bioconductor package for the analysis of Infinium DNA methylation microarrays. Bioinformatics.

[29] Du P, Zhang X, Huang CC (2010). Comparison of Beta-value and M-value methods for quantifying methylation levels by microarray analysis. BMC Bioinform.

[30] Reinius LE, Acevedo N, Joerink M (2012). Differential DNA methylation in purified human blood cells: implications for cell lineage and studies on disease susceptibility. PLoS ONE.

[31] Koestler DC, Christensen B, Karagas MR (2013). Blood-based profiles of DNA methylation predict the underlying distribution of cell types: a validation analysis. Epigenetics.

[32] Jaffe AE, Irizarry RA (2014). Accounting for cellular heterogeneity is critical in epigenome-wide association studies. Genome Biol.

[33] Houseman EA, Accomando WP, Koestler DC (2012). DNA methylation arrays as surrogate measures of cell mixture distribution. BMC Bioinform.

[34] Kim D, Langmead B, Salzberg SL (2015). HISAT: a fast spliced aligner with low memory requirements. Nat Methods.

[35] Li H, Handsaker B, Wysoker A (2009). The sequence alignment/map format and SAMtools. Bioinformatics.

[36] Anders S, Pyl PT, Huber W (2015). HTSeq—a Python framework to work with high-throughput sequencing data. Bioinformatics.

[37] Vanderwall C, Eickhoff J, Randall Clark R, Carrel AL (2018). BMI z-score in obese children is a poor predictor of adiposity changes over time. BMC Pediatr.

[38] Nagin DS (1999). Analyzing developmental trajectories: a semiparametric, group-based approach. Psychol Methods.

[39] Nagin DS, Odgers CL (2010). Group-based trajectory modeling in clinical research. Annu Rev Clin Psychol.

[40] Jones BL, Nagin DS, Roeder K (2001). A SAS procedure based on mixture models for estimating developmental trajectories. Sociol Methods Res.

[41] Ray MA, Tong X, Lockett GA, Zhang H, Karmaus WJ (2016). An efficient approach to screening epigenome-wide data. Biomed Res Int.

[42] Baron RM, Kenny DA (1986). The moderator-mediator variable distinction in social psychological research: conceptual, strategic, and statistical considerations. J Pers Soc Psychol.

[43] James LR, Brett JM (1984). Mediators, moderators, and tests for mediation. J Appl Psychol.

[44] Hallquist MN, Wiley JF (2018). MplusAutomation: an R package for facilitating large-scale latent variable analyses in Mplus. Struct Equ Model.

[45] Fraser A, Macdonald-Wallis C, Tilling K (2013). Cohort profile: the Avon longitudinal study of parents and children: ALSPAC mothers cohort. Int J Epidemiol.

[46] Boyd A, Golding J, Macleod J (2013). Cohort profile: the 'children of the 90s'—the index offspring of the Avon longitudinal study of parents and children. Int J Epidemiol.

[47] Healthy weight, nutrition, and physical activity: about child and teen BMI. 2021. https://www.cdc.gov/healthyweight/assessing/bmi/childrens_bmi/about_childrens_bmi.html#.

[48] Mortensen LJ, Kreiner-Møller E, Hakonarson H, Bønnelykke K, Bisgaard H (2014). The PCDH1 gene and asthma in early childhood. Eur Respir J.

[49] Koppelman GH, Meyers DA, Howard TD (2009). Identification of PCDH1 as a novel susceptibility gene for bronchial hyperresponsiveness. Am J Respir Crit Care Med.

[50] Yoon SY, Shin ES, Park SY (2016). Association between polymorphisms in bitter taste receptor genes and clinical features in Korean asthmatics. Respiration.

[51] Rathod A, Duan J, Zhang H (2020). Interweaving between genetic and epigenetic studies on childhood asthma. Epigenet Insights.

[52] Patil VK, Holloway JW, Zhang H (2013). Interaction of prenatal maternal smoking, interleukin 13 genetic variants and DNA methylation influencing airflow and airway reactivity. Clin Epigenet.

[53] Farahi N, Paige E, Balla J (2017). Neutrophil-mediated IL-6 receptor trans-signaling and the risk of chronic obstructive pulmonary disease and asthma. Hum Mol Genet.

[54] Sindhu S, Thomas R, Shihab P, Sriraman D, Behbehani K, Ahmad R (2015). Obesity is a positive modulator of IL-6R and IL-6 expression in the subcutaneous adipose tissue: significance for metabolic inflammation. PLoS ONE.

[55] Revez JNMA. The role of the interleukin-6 pathway in asthma. PhD Thesis, The University of Queensland; 2018.

[56] Fradin D, Boelle PY, Belot MP (2017). Genome-wide methylation analysis identifies specific epigenetic marks in severely obese children. Sci Rep.

[57] Pinnick KE, Nicholson G, Manolopoulos KN (2014). Distinct developmental profile of lower-body adipose tissue defines resistance against obesity-associated metabolic complications. Diabetes.

[58] Albuquerque D, Nobrega C, Manco L, Padez C (2017). The contribution of genetics and environment to obesity. Br Med Bull.

[59] Dorajoo R, Ong RT, Sim X (2017). The contribution of recently identified adult BMI risk loci to paediatric obesity in a Singaporean Chinese childhood dataset. Pediatr Obes.

[60] Wu K, Gamazon ER, Im HK (2014). Genome-wide interrogation of longitudinal FEV1 in children with asthma. Am J Respir Crit Care Med.

[61] Ranganathan P, Pramesh CS, Buyse M (2015). Common pitfalls in statistical analysis: Clinical versus statistical significance. Perspect Clin Res.

