# Supplementary material for: BMI trajectory in childhood is associated with asthma incidence at young adulthood mediated by DNA methylation
Source: Allergy Asthma Clin Immunol. 2021 Jul 23;17:77. doi: 10.1186/s13223-021-00575-w (PMC8299682; doi:10.1186/s13223-021-00575-w)
Supplement: Supplementary file 1 — Additional file 1: Table S1. Distribution of variables in each of the BMI trajectory groups. Figure S1. BMI trajectories across first 7 years of life in boys and girls respectively in ALSPAC. [file 13223_2021_575_MOESM1_ESM.docx]

Additional Table S1: Distribution of variables in each of the BMI trajectory groups

| **Variables** |  | **Males (n=602), n (%)** | | **Females (n=577), n (%)** | |
| --- | --- | --- | --- | --- | --- |
| Categorical variables |  | Normal BMI trajectory n=547 (88.5%) | High BMI trajectory n=55 (11.5%) | Normal BMI trajectory n=479 (82.1%) | High BMI trajectory n= 98 (17.9%) |
| Asthma incidence | Yes | 9 (81.82%) | 2 (18.18%) | 4 (57.14%) | 3 (42.86%) |
|  | No | 92 (87.62%) | 13 (12.38%) | 72 (81.82%) | 16 (18.18%) |
| Socio-economic status | Low | 13 (92.86%) | 1 (7.14%) | 15 (93.75%) | 1 (6.25%) |
|  | Mid | 78 (84.78%) | 14 (15.22%) | 56 (78.87%) | 15 (21.13%) |
|  | High | 9 (100%) | 0 (0%) | 5 (62.50%) | 3 (37.50%) |
| Active smoking status (18 years) | Past | 22 (84.62%) | 4 (15.38%) | 16 (80%) | 4 (20%) |
|  | Current | 27 (96.43%) | 1 (3.57%) | 14 (82.35%) | 3 (17.65%) |
|  | Never | 51 (83.61%) | 10 (16.39%) | 46 (80.70%) | 11 (19.30%) |
| Second-hand smoking (1, 2, 4 years) | Yes | 52 (86.67%) | 8 (13.33%) | 43 (82.69%) | 9 (17.31%) |
|  | No | 48 (88.89%) | 6 (11.11%) | 33 (76.74%) | 10 (23.26%) |
| Continuous variables |  | Mean ± SD | | Mean ± SD | |
| Age of puberty |  | 14.15 ± 1.14 | 14.13 ± 0.74 | 12.68 ± 1.30 | 12.35 ± 1.54 |


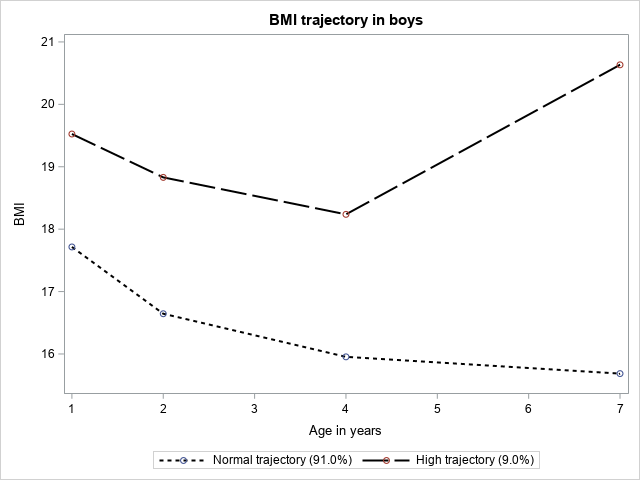

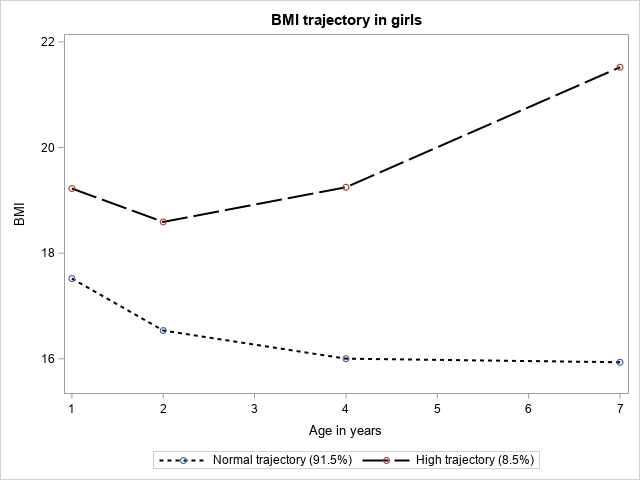


Additional Figure S1: BMI trajectories across first 7 years of life in boys and girls respectively in ALSPAC
